# Workflow for Statistical Analysis of Environmental Mixtures

**DOI:** 10.1021/EHP.6c00155

**Published:** 2026-04-19

**Authors:** Bonnie R. Joubert, Glenn Palmer, David Dunson, Marianthi-Anna Kioumourtzoglou, Brent A. Coull

**Affiliations:** † National Institute of Environmental Health Sciences, National Institutes of Health, Durham, North Carolina 27709, United States; ‡ Department of Statistical Science, 3065 Duke University, Durham, North Carolina 27708, United States; § Department of Environmental Health Sciences, Columbia University Mailman School of Public Health, New York, New York 10032, United States; ∥ Department of Biostatistics, Harvard T.H. Chan School of Public Health, Boston, Massachusetts 02115, United States

## Abstract

**BACKGROUND**: Human exposure to complex, changing, and variably correlated mixtures of environmental chemicals has presented analytical challenges to epidemiologists and human health researchers. There has been a wide variety of recent advances in statistical methods for analyzing mixtures data, with most methods having open-source software for implementation. However, there is no one-size-fits-all method for analyzing mixture data given the considerable heterogeneity in scientific focus and study design. For example, some methods focus on predicting the overall health effect of a mixture and others seek to disentangle main effects and pairwise interactions. Some methods are only appropriate for cross-sectional designs, while other methods can accommodate longitudinally measured exposures or outcomes. **OBJECTIVES**: This article focuses on simplifying the task of identifying which methods are most appropriate to a particular study design, data type, and scientific focus. **METHODS**: We present an organized workflow for statistical analysis considerations in environmental mixtures data and two example applications implementing the workflow. This systematic strategy builds on epidemiological and statistical principles, considering specific nuances for the mixtures’ context. We also present an accompanying methods repository to increase awareness of and inform application of existing methods and new methods as they are developed. **DISCUSSION**: We note several methods may be equally appropriate for a specific context. This article does not present a comparison or contrast of methods or recommend one method over another. Rather, the presented workflow can be used to identify a set of methods that are appropriate for a given application. Accordingly, this effort will inform application, educate researchers (e.g., new researchers or trainees), and identify research gaps in statistical methods for environmental mixtures that warrant further development.

## Introduction

Human exposure to complex mixtures of environmental chemicals has presented analytical challenges to epidemiologists and human health researchers more broadly. However, through recent advances from the statistical and quantitative communities, a wide variety of statistical methods for analyzing mixtures data now exist. In fact, in the last ten years, over 50 statistical methods for environmental mixtures analysis have been published, in part in response to the unmet needs noted in the 2015 National Institute of Environmental Health Sciences (NIEHS) workshop on statistical methods for mixtures,^1^ the 2017 NIEHS Powering Research through Innovative Methods for mixtures in Epidemiology (PRIME) program,^2^ and through investigator-initiated research projects supported by institutions or other US and international funding agencies. Most methods include open-source software for implementation, with accompanying vignettes or instructional documentation and simple example data sets. Although there remains a need for new methodology, the field has come a long way and now includes a rich collection of statistical methods available for analyzing mixture data from a variety of study designs (cross sectional, longitudinal exposures and/or outcome, time-to-event, etc.) and for a range of inferential interests (prediction of the overall health effect of the mixture, inference on which constituents significantly impact health, estimation of main effects and interactions, etc.).

The collection of available methods and considerations for their applications has been described in several reviews.^3–9^ To guide researchers navigating informed applications, methods are often organized by the research question(s) addressed. Braun et al.^3^ present three key research questions, expanded to four by Hamra et al.:^6^ (a) What is the effect of an aggregate mixture? (b) What is the effect of a sum of mixture components? (c) What are the independent effects of mixture components? and (d) What are the joint effects of mixture components? Gibson et al.^5^ present a slight variation of these questions, tailored to an analysis data set, reiterated by Joubert et al.,^9^ as methods designed to conduct: (a) overall effect estimation, (b) toxic agent identification (variable selection), (c) pattern identification, (d) health effects estimation in the presence of *a priori* defined groups, and (e) estimation of interactions and nonlinearities. Some reviews have also organized mixtures methods into the two broad categories of supervised and unsupervised learning methods,^5^ the latter focused on the exploration of patterns in exposure data, independent of a specific outcome or health end point. Although these guidelines organize methods into well-defined categories, it is common for one method to address more than one research question, and several methods may be appropriate in any given context. In addition to research questions and study design, researchers deciding between various methods also need to consider the distribution of and measurement of the outcome (continuous, binary, time-to-event, repeated measures etc.), the structure of the measured exposure data (e.g., varying in space and/or time), the size of the data set, and other considerations.

Understandably, researchers may incorporate individual preferences in methods selection reflecting their training, recommendations from statistical collaborators, interpretability of the results, access to published methodology (open access publications), open access software, or other factors. Clear and freely available instructions ranging from minimal documentation accompanying *R* packages in *GitHub* to more detailed vignettes in CRAN or in eBooks^10^ and inclusion of methods in institutional coursework or short training programs such as the Columbia SHARP Mixtures Workshop^11^ can ideally facilitate the new use and reuse of available methods. Nonetheless, researchers, particularly new researchers, may find it difficult to know where to start, which methods to select and why, and how many methods are applicable for any given analysis. As such, informed methods selection remains an issue for environmental health researchers when designing a study, dissertation or thesis project, or grant application, or when responding to peer review of a publication.

To date to our knowledge, no courses or texts have presented a systematic approach to selecting a mixtures method, including how features of the data and study design inform this choice. This gap in the literature motivated our work to develop a workflow that organizes mixtures methods into meaningful categories, guided collectively by the scientific question of interest, the design of the study that generated the data, and the resulting structure of the available data. The workflow can be used to inform application, facilitating method selection, and increase awareness of new methods. Importantly, instead of comparing methods in a competitive way, offering one as more or less preferable than another, this workflow offers an agnostic guide that facilitates leveraging multiple methods for a variety of contexts.

## Workflow for Conducting Environmental Mixtures Analysis

We present an organized series of steps (here, referred to as a workflow) for approaching environmental mixtures analysis of epidemiological data. The goal is to provide a guide for researchers having a particular data set and inferential focus in hand. The steps in the workflow are intended to be followed sequentially, starting with the overall conceptual model for the analysis of interest (Step 1) and data processing and exploratory analyses (Step 2). Information regarding the study design and data characteristics (Step 3), existing scientific knowledge (Step 4), and the specific research questions of interest (Step 5), can be assessed in any order and will result in a list of relevant statistical models that can be applied to address a particular mixtures analysis context or hypothesis. The final Step 6 includes considerations for model assessment and evaluation. The workflow is summarized in Figure 1 with additional text in Supplementary Table 1. The presented workflow builds on the available resources to date and covers key questions based on methodological principles of epidemiology, tailored to the context of environmental mixtures.

**Figure 1:**
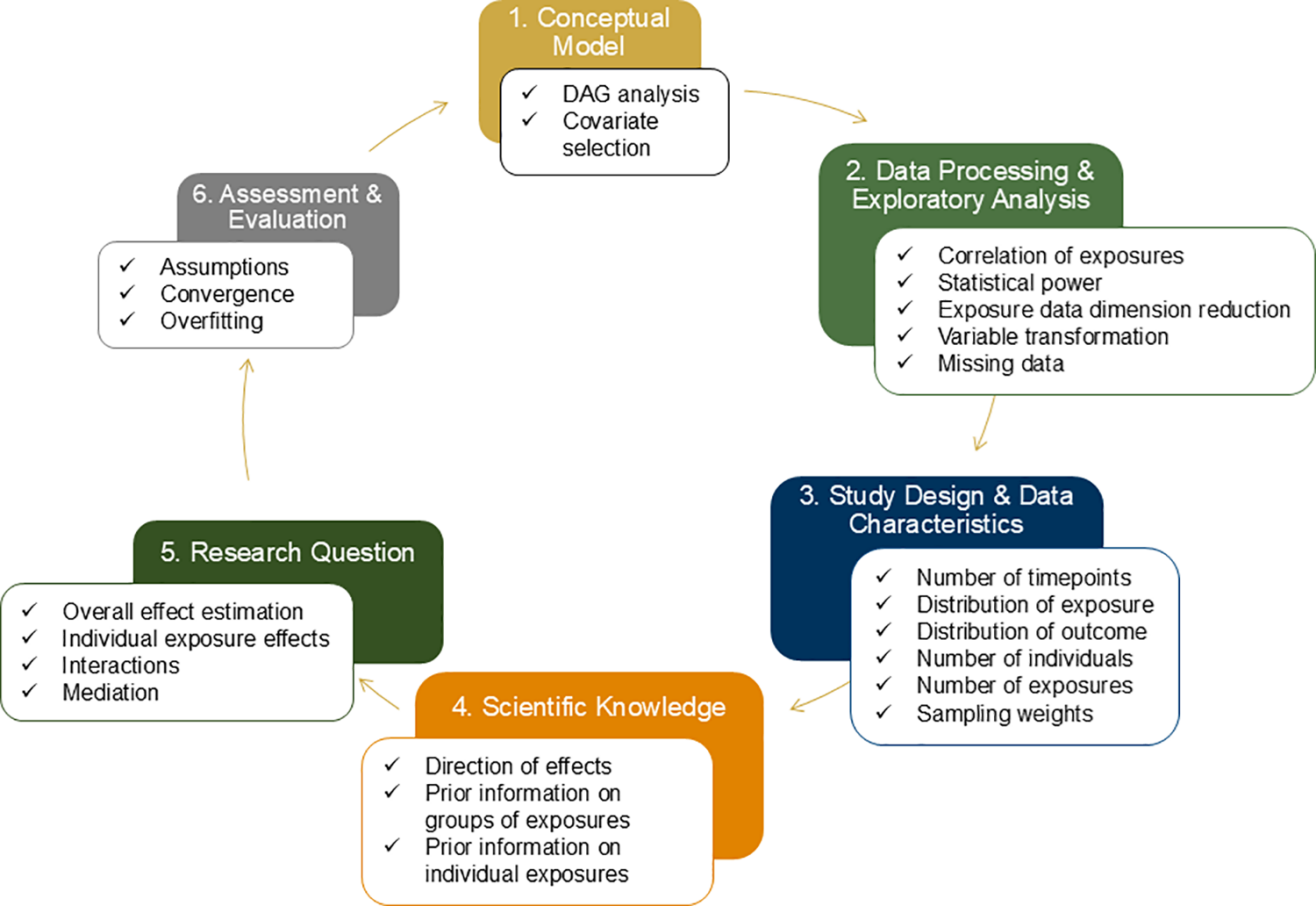
Summary of mixtures method selection workflow steps 1–6.

## Step 1. Conceptual Model Development

### Directed Acyclic Graph Analysis and Covariate Selection

To hypothesize the causal relationships between the outcome and exposures considered to be part of the mixture, as well as covariates of interest, researchers can apply Directed Acyclic Graph (DAG) analysis. Examples of standard DAG analysis in epidemiology have been previously described (Lash et al., Modern Epidemiology, fourth edition).^12^ Covariate selection should also be carefully considered, including whether a covariate is associated with both the exposure(s) and the outcome of interest, whether it operates as a modifier of the exposures-outcome association, or whether it should be considered as a mediator. For the mixtures context, Weisskopf et al.^13^ describe example scenarios where including multiple correlated variables in a model such as a regression can increase (amplify) bias in the model, particularly when using variables representing biomarkers of environmental exposures. This bias is referred to as coexposure amplification bias (see the Glossary in the Supplementary Text), and is important to consider in early stages of mixtures analysis. Overall, the conceptual model development phase should result in a list of variables of interest and an analysis data set to which a mixtures analysis can be applied. In this design phase, researchers should also consider selection/collider bias. It is important to remember that the study DAG may vary depending on the research question of interest (i.e., whether the study evaluates overall effects, individual exposure effects, or effect mediation).

## Step 2. Data Processing and Exploratory Analysis

### Examine Correlation of Exposures

A key step in the analysis of environmental mixtures data is to explore the structure and magnitude of correlations among exposure variables. This exploratory analysis is helpful for several reasons. First, it can help inform which methods will be effective for a given data set. For instance, if correlations among a set of exposures are 0.9 or above, it could be the exposures arise from the same source and almost always travel together, making it difficult for any mixture model to untangle the effects of individual exposures, especially if the sample size is small. In such cases, it may be more useful to employ careful dimension reduction prior to modeling the exposures or to use a grouped variable selection method that quantifies the importance of the group of variables as a whole in the model. Knowledge of the magnitudes of the correlations can also help one understand what exposure scenarios, and therefore what contrasts of the mixture, are realistic. For instance, if two exposures have a pairwise correlation of 0.8, then it is unlikely that there are any subjects in the data set having low levels of one exposure but high values for the other. In this case, predicting the mean outcome under a low-high exposure scenario would extrapolate outside of the observed data. If pairwise correlations are low, there may not be many observations with extremely high levels of all exposures (i.e., all at 90% percentile). Antonelli and Zigler also emphasize the importance of understanding whether exposure contrasts are observable within a given data set or require extrapolation.^14^

### Examine Statistical Power

Before performing analysis, it may be useful to investigate the probability that a given method will detect an association given an assumed data generation model, sample size, and effect size. This probability is referred to as statistical power and is particularly important in planning a study, as it can guide what sample size is needed, but can also be useful in selecting a model based on an assumed data generating mechanism and effect size. While closed-form formulas are available for power in very simple settings, complex data like in the mixtures setting generally require a simulation study. To facilitate this, the *mpower* *R* package implements simulation-based power estimation specifically for the mixtures setting.^15^

### Exposure Data Dimension Reduction (Optional)

It is possible, especially with highly correlated ambient exposures such as air pollution, that researchers may want to first identify patterns in the exposure space (e.g., air pollution sources) and reduce the dimension of the mixture prior to health analyses. Several methods can be used without including information about the health outcome (unsupervised methods). Example methods appropriate for mixtures data include Principal Components Pursuit (PCP)^16^ or Bayesian Factor Analysis.^17^ This step is considered optional, depending on the exposure data characteristics. These methods can also be used for exposure pattern recognition, a mixtures-related research question that we do not cover as part of this seminar paper.

### Variable Transformation (Optional)

Another optional step prior to data analysis is to transform and scale the outcome, exposure, and/or covariate variables. It is important to standardize the exposure variables, so they are all on the same scale. Because some popular mixtures models assume normality for a continuous outcome, it can be helpful to log-transform such outcomes if they exhibit a log-normal distribution (see Step 3, Distribution of the Outcome). We consider these as standard data preparation steps, part of core graduate level curriculum for epidemiology and biostatistics. Some mixtures methods are based on a particular type of transformation of exposure data, such as weighted quantile sum regression and quantile G-computation, which use quantiles of the exposure distributions by default. Example code for variable selection and transformation steps are available in the Columbia Mixtures Workshop GitHub (https://github.com/lizzyagibson/SHARP.Mixtures.Workshop).

### Manage Missing Data

A critical step in data processing is to address missing data. Missing data can occur in several forms, such as information indicating an exposure is below the limit of detection (LOD) or an exposure value is simply not available. To our knowledge, only a few mixtures methods formally incorporate missing values into a unified modeling framework. Herring^18^ proposed a nonparametric Bayes approach to modeling exposures and health outcomes when the exposures are subject to limits of detection. More recently, Ferrari and Dunson^19^ proposed Bayesian factor analysis for inference on interactions. Because both the observed exposures and outcomes are jointly modeled by latent factors (see Glossary), this method can have missing exposure values as inputs into the method. Bayesian profile regression is another example of a Bayesian model that jointly models the distributions of the exposure and outcome data, and therefore able to accommodate exposure data below the LOD although to the best of our knowledge has not been used for this purpose to date.^20–22^ Otherwise, most mixtures methods do not allow the inclusion of missing values, so rows with missing data must be removed, or missing data imputed. This typically applies to both supervised and unsupervised methods, although some unsupervised methods such as PCP^16^ can also handle missing data and values below the LOD.

If exposure data are subject to limits of detection,^23^ popular strategies such as replacing a value below the LOD divided by √2 can be used, but yield biased health effect estimates. Lee et al.^24^ examined common LOD accommodation approaches on mixture analysis results where multiple exposures are below the LOD: complete case analysis, single imputations by LOD/√2 or from a censored accelerated failure time (AFT) model; and multiple imputation (MI) with or without truncation based on LOD. They examined this in three mixture models: elastic net regression, weighted quantile sum (WQS) regression, and BKMR. Through both simulation and using data from the National Health and Nutrition Examination Survey (NHANES), notably the same NHANES example data we apply here in later example application of the workflow, they observed unstable performance of imputation by LOD divided by √2 and favored use of truncated multiple imputation and censored accelerated failure time models.

More generally (not just for handling exposure values below LOD), multiple imputation is an approach that generates multiple realizations of missing exposure data from an assumed model and then uses the multiple copies of the data in subsequent health effects analyses to account for the uncertainty induced by the missingness. The resulting multiple values of health effect estimates can then be combined to produce an overall effect estimate, and associated uncertainty, using rules developed by Rubin.^25^ Lubin et al.^23^ described how this can be done using a log-normal model for an exposure distribution, and the experimental package *censlm* implements this approach (https://github.com/mikmart/censlm). One can also use multiple imputation to generate multiple realizations of exposure values missing more generally, rather than below the LOD. A popular approach for this task is Multiple Imputation by Chained Equations (MICE),^26^ with a robust *R* package for implementation.^27^ In frequentist mixtures analyses, such as weighted quantile sum (WQS) regression or quantile G-computation, one can use MICE to obtain multiple realizations of missing values for multiple exposures, feed each resulting copy of the data into the mixtures model, and combine the resulting effect estimates using Rubin’s rule. This approach assumes the data are “missing at random”; that is, the missingness mechanism depends solely on data that are observed, not on unobserved factors. While a formal, rigorous Bayesian approach would jointly fit the exposure imputation model and health model jointly, as in Herring,^18^ Ferrari and Dunson,^19^ and other work, Bauer et al.^28^ approximated this approach by applying a Bayesian health model (BKMR, in this case) to each realization of data obtained by MICE and accounted for the resulting uncertainty by amalgamating the posterior samples from each run into a single posterior sample. Code for implementing this approach using BKMR is available at https://github.com/kdevick/bkmr_MI.

## Step 3. Study Design and Data Characteristics

Following the conceptual design and data examination and processing steps, researchers can then address questions relevant to the epidemiologic study design and variable characteristics of their analysis data set, as not all mixtures methods are suitable for all design/data contexts. Specifically, researchers should consider whether the design is longitudinal and includes measurements of exposures and/or outcomes at multiple time points and whether spatial data are examined. Researchers should also consider the distribution of the outcome and size of the data set and may wish to run through these steps more than once, for different variable transformations (e.g., evaluating a continuous outcome in one analysis, dichotomous outcome in a separate analysis). Steps 3–6 are presented as yes/no (0/1) prompts for each method listed in Figure 2 and can be addressed in any order.

**Figure 2:**
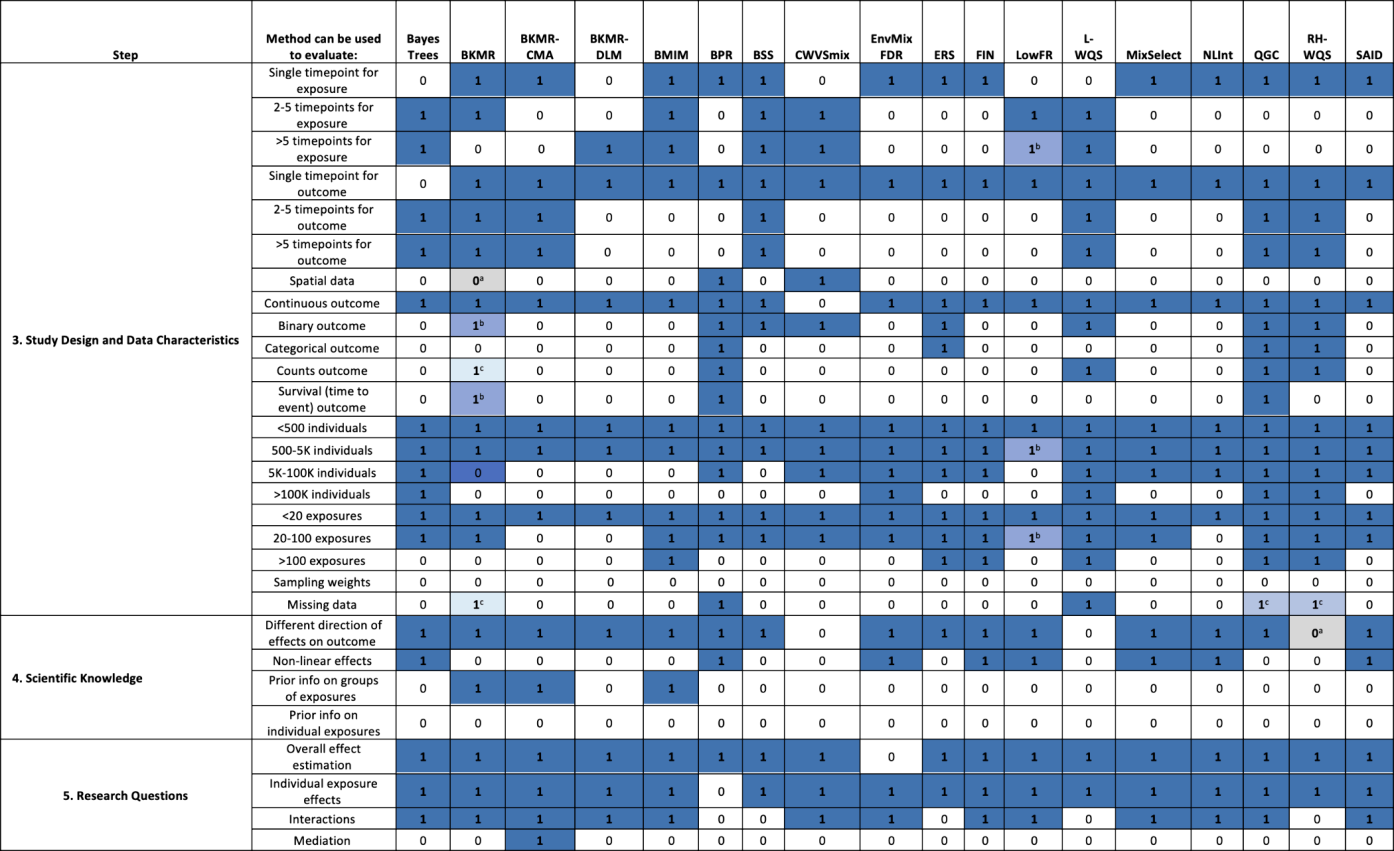
Summary of the Mixture Method Selection Workflow Steps 3–5 for Example Statistical Methods 0 = No/1 = Yes responses reflect how the method was described in the original publication or in publications applying the method in an epidemiological data set and where publicly available code with documentation (e.g., an *R* package) for this type of application is available. 0^a^ : It is possible to manipulate the source code to align with this feature (e.g., using a cox proportional hazards partial likelihood to perform conditional logistic regression), but the code is not currently publicly available. 1^b^ : The method can be applied but may have lower performance/speed or interpretability compared to other methods. 1^c^ : This method can be applied with publicly available code that is outside the main methods package. Methods: Bayes Tree Pairs (BayesTrees); Bayesian Kernel Machine Regression (BKMR); BKMR-Causal Mediation Analysis (BKMR-CMA); BKMR-Distributed Lag Model (BKMR-DLM); Bayesian Multiple Index Models (BMIM); Bayesian Profile Regression (BPR); Bayesian Subset Selection (BSS); Critical Window Variable Selection for Mixtures (CWVSmix); Environmental mixtures with FDR control (EnvMixturesFDR); Environmental Risk Score (ERS); Factor Analysis for Interactions (FIN); Low-rank longitudinal factor regression (LowFR); Lagged Weighted Quantile Sum Regression (LWQS); Identifying main effects and interactions among exposures using Gaussian processes (MixSelect); Bayesian semiparametric regression with sparsity inducing priors (NLInt); Quantile G computation (QGC); WQS and Repeated Holdout WQS (RH-WQS); Synergistic and antagonistic interactions in mixtures of exposures (SAID).

### Single or Repeated Time Points of Exposures and Outcomes

For this design/data characteristic, researchers can address the question: “Is the study longitudinal, with repeated measurements of exposures and/or outcomes or is there only a single timepoint for the measurement of exposure and outcome variables?” Historically, most methods for mixtures analyses have not considered multiple time points of exposure and outcome measurements. Now several models exist that can leverage longitudinal data and one can consider whether or not the analysis data set includes repeated measurements of exposures and/or outcomes, and, if so, the number of time points of interest to include in a model. Often this requires considering a distributed lag model (DLM, see Glossary) or other methods that account for repeated time points. For example, CWVSmix, BKMR-DLM, Bayesian Tree Pairs, and Lagged WQS were designed to handle relatively many repeated, regularly measured values of multiple exposures (e.g., weekly measures of ambient air pollutants or temporally resolved tooth biomarkers of exposure) (Table 1). CWVSmix and BKMR-DLM assume smoothly varying effects of each chemical across pregnancy, so are limited to such designs, whereas Bayesian Tree Pairs, which uses tree-based methods to identify windows of susceptibility, could conceivably be applied to exposure data measured on fewer time points (e.g., trimester-specific measures). Development of methods that can address both big data and longitudinal data is one area that may warrant further research.

**Table 1:** Summary of Example Mixtures Methods Presented for the Workflow^a^

**Method**	**Summary**	**Reference**	**Code**
Bayes Tree Pairs (BayesTrees)	Considers a situation like BKMR-DLM but uses tree-based models to identify critical windows of exposure for a mixture. Scalable to larger data sets as compared to BKMR-DLM.	Mork and Wilson^51^	https://github.com/danielmork/dlmtree
Bayesian Kernel Machine Regression (BKMR)	Models a real-valued or binary outcome on a set of exposures using a potentially nonlinear exposure-response function modeled by a kernel function. Can accommodate repeated outcome measures using random subject-specific effects.	Bobb et al.^52^	https://rdocumentation.org/packages/bkmr/versions/0.2.2
BKMR-Causal Mediation Analysis (BKMR-CMA)	Performs a causal mediation analysis when interest focuses how the effect of a exposure mixture on a real-valued outcome is mediated through a real-valued mediator	Devick et al.^38^	https://github.com/kdevick/BKMR-CMA
BKMR-Distributed Lag Model (BKMR-DLM)	Applies when interest focuses on identifying critical windows of exposure for each of multiple exposures within a mixture on a real-valued outcome, and repeated data on each exposure are available on a regular grid (such as weekly during pregnancy). Uses a distributed lag model (DLM) formulation within a Bayesian kernel machine formulation for the exposure.	Wilson et al.^1000^	https://github.com/niehs-prime/regimes
Bayesian Multiple Index Models (BMIM)	Models a real-valued outcome on multiple groups of exposures by assuming group-specific linear combinations of exposures are inputs into a multivariate exposure-response surface.	McGee et al.^41^	https://github.com/glenmcgee/BMIM
Bayesian Profile Regression (BPR)	Uses a profile formed from a sequence of covariate values, clustered into groups and associated via a regression model to a relevant outcome.	Molitor et al.^22^	CRAN: Package PReMiuM (r-project.org)
Bayesian Subset Selection (BSS)	Can select variables and provide uncertainty quantification for any linear Bayesian model and identify variables to include in subsequent steps.	Kowal et al.^53^	https://github.com/drkowal/BayesSubsets
Critical Window Variable Selection for Mixtures (CWVSmix)	An extension of the “Critical Window Variable Selection” model of Warren et al. (2020)^36^ to the mixtures setting.	Wilson et al.^54^	https://github.com/warrenjl/CWVSmix
Environmental mixtures with FDR control (EnvMixturesFDR)	Simultaneously estimates the health effects of environmental mixtures and identifies important exposures and interactions while controlling FDR.	Samanta and Antonelli^55^	https://github.com/srijata06/EnvMixturesFDR
Environmental Risk Score (ERS)	Integrates information on the individual exposure-health outcome effects for multiple exposures by estimating a combined risk score, accounting for covariates, on a portion of the data, and then tests for an association between this risk score and the outcome in the remaining data.	Park et al.^50^	https://github.com/um-mpeg/Environmental-Risk-Score
Factor Analysis for Interactions (FIN)	Models a real-valued outcome using a quadratic regression on a cross-sectional mixture of exposures.	Ferrari et al.^19^	https://github.com/niehs-prime/factor_interactions
Low-rank longitudinal factor regression (LowFR)	Models a real-valued outcome using a quadratic regression on all measurements of a longitudinally measured mixture of exposures. Best suited for a relatively small number of measurement times (as opposed to some of the DLM approaches above that require a larger number of measurement times).	Palmer et al.^56^	https://github.com/glennpalmer/lowfr
Lagged Weighted Quantile Sum (L-WQS) Regression	Assesses susceptible windows of exposure to mixtures by estimating a joint multipollutant effect at each time point.	Gennings et al.^57^	https://cran.r-project.org/web/packages/gWQS/index.html
Identifying main effects and interactions among exposures using Gaussian processes (MixSelect)	Models a real-valued outcome using a nonlinear regression on a cross-sectional mixture of exposures.	Ferrari et al.^40^	https://github.com/fedfer/MixSelect
Bayesian semiparametric regression with sparsity inducing priors (NLInt)	Models the effect of an interaction of exposures within a mixture on a continuous outcome, where the form of the multivariate exposure-response surface is potentially complex.	Antonelli et al.^58^	https://github.com/jantonelli111/NLinteraction
Quantile G computation (QGC)	Constructs an index of exposures and estimates an overall effect of this index. Does not assume directional homogeneity of effects. Applies to a real-valued, binary, multinomial, count, or survival outcome.	Keil et al.^59^	CRAN: Package *qgcomp* (r-project.org)
Repeated Holdout Weighted Quantile Sum (RH-WQS) Regression	Constructs a linear-index of the exposures and estimates an overall effect of the index. Assumes the exposures are associated with the outcome in the same direction (directional homogeneity), although recent extensions allow for multiple indices capturing effects in both directions. Applies to a real-valued, binary, multinomial, count, or zero-inflated count outcome.	Tanner et al.^46^	CRAN: Package *gWQS* (r-project.org); https://github.com/evamtanner/Repeated_Holdout_WQS
Synergistic and antagonistic interactions in mixtures of exposures (SAID)	Finds nonlinear interaction surfaces for synergistic and antagonistic pairwise interactions.	Chattopadhyay et al.^60^	https://github.com/shounakch/SAID

a Methods include statistical methods for environmental mixtures, findable in PubMed, Web of Science, Embase, or Scopus, guided by the following criteria: 1) designed to examine the association between multiple (at least three) environmental exposure variables and a human health outcome; 2) published in the last ten years (January 1, 2015, or later); 3) published with findable open-source software such as an *R* package posted in *GitHub* with sufficiently detailed software documentation to inform application, and 4) sufficiently within the expertise of the authors so we could confidently assess each step in the workflow. We also considered methods described in review articles published in the last five years (January 1, 2020, or later) on mixtures methods, prioritizing methods from other research groups. Methods addressing computational toxicology, risk assessment, exposure prediction, or focused on nonchemical/environmental exposures were considered out of scope.

### Spatial Data

For this design/data characteristic, researchers can address the question: “Does the study include data with spatial variation and/or does spatial correlation among outcomes need to be considered in the model?” Studies have demonstrated the importance of fine particulate matter (PM_2.5_) on health outcomes, including cardiorespiratory and neurological outcomes, and that the effects can be spatially heterogeneous.^29^ Importantly, the composition of particulate matter includes a mixture of chemicals including elemental carbon, arsenic, sulfate, silicon, and metals such as lead, iron, manganese, and zinc. As such, researchers working with air pollution data or other data that are spatially heterogeneous may need mixtures models that can specifically address spatial heterogeneity. In addition to air pollution data, researchers may also wish to consider meteorological or geographical factors such as wind speed, ambient temperature, or humidity.

For data sets with spatial correlation in the exposure data varying in space and time (e.g., air pollution composition data), researchers also need to consider estimation of parameters that may vary in space and time both in direction and magnitude. Jin et al.^30^ proposed such an approach, leveraging the role of wind direction on air pollution spread, while using Bayesian methods to allow for uncertainty in the directional DAG. Their “bag of DAGs” methodology speeds up computation for large spatiotemporal data sets relative to popular Gaussian process random effects approaches, while accommodating a more flexible covariance structure.

When there is residual spatial correlation in the outcome, ideally this would be modeled with spatially correlated random effects.^31^ While there are relatively few mixtures methods that allow for spatial correlation among random effects, an exception is work by Mutiso et al.^32^ for small-area disease rates. This work modeled spatial correlation among areal disease counts using conditionally autoregressive random effects. Researchers interested in similar applications can contact the manuscript authors for example code.

### Distribution of the Outcome

For this design/data characteristic, researchers can address the question: “Does the dataset include a continuous, binary, categorical, count, or time-to-event (survival) outcome variable?” While most of the strategies underlying specific methods could conceivably be extended to many of these outcome types, typically the software accompanying a particular method only handles certain types of outcomes. Some methods, such as BKMR, Bayes Trees Pairs, FIN, and MixSelect were developed for analysis using a continuous outcome variable (Table 1). The *bkmr* *R* package also implements BKMR using a probit link for binary outcomes, and Generalized BKMR^33^ can be implemented using the *R* package *gbkmr* (https://github.com/abc1m2x3c/GBKMR) for continuous, binary, and count outcomes. Other methods such as CWVSmix are suitable for binary outcomes but not continuous, categorical, counts, or survival outcomes, whereas QGC and WQS are appropriate for binary, categorical, and continuous outcomes. QGC can also handle survival (time to event) outcomes. For models that assume normality for the residuals of a model using a continuous outcome, it can be helpful to log-transform such outcomes to improve the normality assumption for the model errors.

### Size of the Data Set

The size of a data set is commonly described as the number of individuals and the number of variables/exposures of interest to include in a specific model. Thus, for this design/data characteristic, researchers can address the question: “How many individuals and how many exposures/variables are included in the dataset for analysis?” For extremely large data sets, such as administrative data, applying a mixtures model that is not suitable for big data can result in extensive computational time and possibly issues with model convergence. To identify the best method, researchers can consider whether an analysis includes less than 500, 500–5,000, 5,000–100,000, or over 100,000 individuals and whether the data set includes less than 20, between 20 and 100, or over 100 exposure variables. While multiple methods considered in this workflow handle settings with over 100 variables, we do not consider the broader set of methods designed to analyze data on the exposome, encompassing an individual’s life-course environmental exposures, as there are other reviews of this field available elsewhere.^34,35^ Because many exposomic studies focus on 100s to 1000s of biomarkers of exposure, a popular approach to analysis is an Exposure Wide Association Study (ExWAS), the most common form of which is a discovery-based approach that seeks to identify important phenotype-exposure pairs, while controlling for multiple testing.^34^ In contrast, the mixture methods considered in this workflow focus on one or more of the research questions outlined in Step 5 (Research Questions).

### Survey or Sampling Weights

For this design/data characteristic, researchers can address the question, “Are there survey or sampling weights to include in the analysis?” Many researchers leverage large survey data such as large data from the National Health and Nutrition Examination Survey (NHANES). However, traditional epidemiological models applied to these data apply weights, to account for oversampling strategies. Few mixtures methods can incorporate these weights, which is a known limitation for the field. However, anticipating how methods expand and evolve over time, and given the common use of NHANES data for mixtures analyses, we include this feature as an important component in the workflow.

## Step 4. Scientific Knowledge

It can be helpful to utilize existing information from the literature, preliminary data, and/or toxicology in a model. For example, researchers can consider whether the exposure-outcome association (effect) of each component in a mixture is expected to operate in the same direction on the health outcome, or whether the model should allow effects in different directions. The researcher may also know that the exposure-response relationship is likely to be nonlinear. There may be known structure or *a priori* information about groups of the exposures, such as chemical groups, that should be included in the statistical model. Some methods can leverage this information and group exposures in the modeling (e.g., BKMR hierarchical grouping extension, or Bayesian Multiple Index Models: BMIM). Researchers may also wish to include individual information about exposures in the model, such as biological, toxicological, or other chemical features. For example, BMIM can incorporate the toxic equivalency factor, or related features, of subsets of exposures.^36^ To consider this scientific information, researchers can consider the following questions: “Are exposures hypothesized to act in the same direction, or should the model allow for the possibility effects operate in different directions? Is the exposure-response relationship likely to be nonlinear? Is there biological, toxicological, or other information about the potential effects of the exposures such as chemical groups that should be included in the statistical model? Are there chemical properties/features to include in the model?” Responses to these prompts can be particularly critical to identifying relevant methods, as many existing methods are not able to address all contexts.

## Step 5. Research Question

For this step, researchers can address the question, **“**What is the research question of interest for this analysis?” Identifying the research question and the overall goal of the analysis is critical to mixtures method selection. Although these have been described a few different ways in the literature, questions can roughly align with the following. Of note, these questions are specific to supervised strategies (including both exposure and outcome data). Unsupervised analyses can be considered earlier in the workflow (Step 2).

### Overall Effect Estimation

Researchers often wish to determine the overall or aggregate effect of the mixture of exposures on a health outcome. Some interpret this to represent the effect of a sum of mixture components (separately distinguished in Hamra et al.^6^). Most presented methods can be applied to this research question; however, it is worth noting that the definition of “overall effect” varies method to method. That is, the exposure contrast to which the overall effect corresponds is typically methods specific. Often, a method is selected not for addressing this research question alone, but for being able to address this question as well as one or more other research questions.

### Individual Exposure Effects

Researchers are also often interested in the independent effects of mixture components. This has also been referred to as toxic agent identification or variable selection in Gibson et al^5^ and Joubert et al^9^ and can identify the “toxic agents” or “bad actor(s)” in the mixture of chemical exposures. The majority of methods presented here can also address this research question.

### Interactions

Examining potential interactions is another common goal. Most methods can accommodate user-defined interactions by including products between two exposures as a new mixture member. Among the presented methods here, most methods are able to address interactions when examining a single time point of exposure and outcome. Some methods can also address the effects of interactions within a mixture at multiple time points, including Bayes Tree Pairs, BKMR-DLM, CWVSmix, LowFR, and BMIM (Figure 2).

### Mediation

Examining the effect of a mixture that mediates the association between an exposure and an outcome is a challenging area of mixtures. Bellavia et al.^37^ describe strategies for approaching mediation analysis with environmental mixtures, including multiple regression, reducing the mixture to a single mediator, reducing the number of mediators, hierarchical modeling, and a two-stage approach by using a mixture method to select specific mediators. Devick et al.^38^ present the use of BKMR-Causal Mediation Analysis (BKMR-CMA) for examining the effect of a mixture as an exposure on a health outcome, considering a single mediator. Because this method includes publicly available *R* code for implementation, we include it in the methods presented in Figure 2. Further work with publicly available software for examining the effect of mixtures as the mediator between an exposure and outcome of interest, with publicly available software for implementation, is needed.

## Step 6. Assessment and Evaluation

After considering the above questions, a list of available methods for each research question can be provided as well as context and rationale for these recommendations. For model implementation, it is important to examine results with scrutiny, given the complexities and nuances of mixtures models. Some example assessment considerations are noted here but should not be considered exhaustive.

### Assumptions

It is important to examine underlying assumptions of each model identified relevant for a specific scenario. This is best considered prior to selecting and implementing a particular method. Many of the methods assume residuals in a model for continuous outcomes that are normally distributed with constant variance. Other models may have more specific assumptions. For example, LowFR assumes the exposures can be modeled with a multivariate normal distribution, and the expected outcome is well-modeled as a quadratic function of the exposures. Assumptions for each model should be carefully considered in model selection and interpretation of results.

### Convergence

Several existing mixture methods employ Bayesian methods for modeling fitting and inference. The Bayesian framework characterizes uncertainty in model parameters by conceptualizing them as random variables, and inference proceeds by summarizing a probability distribution for them, known as the posterior distribution, that reflects what values are more and less plausible, given the data. For many models, the posterior distribution of the model parameters is often not available in closed-form but samples from it can be generated using a stochastic Markov Chain process. This process, known as Markov Chain Monte Carlo (MCMC), typically takes multiple sequential samples to reach equilibrium and sample from the actual posterior distribution, also known as MCMC convergence (see the Glossary in the Supplementary Text). It is essential in applied Bayesian statistics to diagnose whether an MCMC algorithm has converged, so that one is confident that the resulting point estimates, credible intervals, and other summary statistics accurately characterize the posterior distribution of the model parameters.

There are multiple options for diagnosing convergence in available Bayesian models for environmental mixtures. A popular approach is to visually assess a plot of the sequential generated posterior samples (trace plot) and assess if these values randomly vary around a stationary mean. One can generate these plots manually using the returned posterior samples. Some software for mixture models include functions for generating these plots automatically and make available tutorials demonstrating how to interpret the resulting plots. See https://jenfb.github.io/bkmr/overview.html for BKMR. Another approach is to run multiple Markov Chains starting from different initial values and assess whether the resulting posterior samples appear to have been generated from the same posterior distribution. The *R* package *coda* can be used for analyzing MCMC output from any model to diagnose convergence. Others have developed packages that provide the ability to similarly run multiple chains and diagnose convergence for specific mixture models, such as *bkmrhat* for BKMR models. No matter the code chosen to diagnose convergence for a given MCMC sampler, this should be standard practice when applying Bayesian models for environmental mixtures data.

### Overfitting

Some methods have model-specific assumptions that should be tested, and each model should be carefully assessed for model fit and performance. For example, a model may appear to fit well to the data used to fit the model, but particularly for highly flexible models, this could simply be a result of interpolation for those specific observations, rather than learning a true underlying relationship. To guard against this, models can be evaluated on “out-of-sample” data. That is, researchers can divide the full data set into nonoverlapping “training data” and “test data” sets, fit the model to only the training set, and then evaluate the quality of predictions of that fitted model on the test data. This can be repeated multiple times with different training/test splits, in a process called cross-validation. The process of evaluating predictions on data not used to fit the model is commonly referred to as evaluating out-of-sample predictive performance (see Glossary in Supplementary Text), and strong performance in this evaluation suggests greater confidence in the inferential conclusions generated by the model.

## Environmental Mixtures Methods Repository

The content of this manuscript can be considered a sufficient stand-alone guide. The workflow offers a clear systematic strategy for approaching mixtures analysis and can be applied to a wide range of mixtures methods, not just those included in the presented tables and text of the manuscript. However, it is expected that new methods and modifications to existing methods will develop over time. As such, we present the NIEHS Environmental Mixtures Methods Repository (https://github.com/NIEHS/emix). This is a NIEHS-managed and sustained GitHub repository (“emix”), where mixtures methods information can be reviewed and updated over time. The repository includes a table listing all methods described in this paper. The table can be tailored to a specific analysis need by using column filters to select features described in Steps 3–5 of the workflow. Researchers can then review details for each method, including the methods paper(s) and software for implementation. The goal of this repository is to enable a sustainable and flexible resource for sharing information about available methods for environmental mixtures in epidemiology, and to provide a useful starting place for new researchers approaching this analysis.

## Methods Considered

For this article, we considered statistical methods for environmental mixtures, findable in PubMed, Web of Science, Embase, or Scopus, guided by the following criteria: 1) designed to examine the association between multiple (at least three) environmental exposure variables and a human health outcome; 2) published in the last ten years (January 1, 2015 or later); 3) published with findable open-source software such as an *R* package posted in *GitHub* with sufficiently detailed software documentation to inform application, and 4) sufficiently within the expertise of the authors so we could confidently assess each step in the workflow. We also considered methods described in review articles published in the last five years (January 1, 2020, or later) on mixtures methods, prioritizing methods from other research groups. Methods addressing computational toxicology, risk assessment, exposure prediction, or focused on nonchemical/environmental exposures were considered out of scope. We summarize 18 methods in Table 1 that best demonstrate the workflow. In addition to the methods considered in this paper, methods meeting criteria 1–3 can be added over time to the NIEHS GitHub Mixtures Methods Repository (https://github.com/NIEHS/emix); we encourage authors of relevant methods to submit them for inclusion.

## Example Applications

### Example 1: National Health and Nutrition Examination Survey (NHANES)

We considered two example scenarios for application and walk through alignment with each workflow step. These are examples of how to employ the workflow, not tutorials on statistical methods themselves—for these details please see the publications and code listed in Table 1. Further, because the purpose of this seminar is not to present data and results, we describe the broad decision-making process and encourage readers to review the links at the end of the paper for access to the publicly available data for more hands-on implementation. We first considered data from the cross-sectional 2001–2002 National Health and Nutrition Examination Survey (NHANES). The NHANES data set with 1,330 adults was previously described and used by Mitro et al.^39^ to examine the association between exposure to 18 persistent organic pollutants (POPs) and leukocyte telomere length (LTL). This data set has been used extensively for training including the Columbia Sharp Mixtures Workshop,^5^ as well as for demonstrating applications of new methods such as FIN^40^ and BMIM.^41^ The original NHANES data and example code for data formatting is available in the Supporting Information of Gibson et al. (https://github.com/lizzyagibson/Mixtures.Workshop.2018).^5^ For the NHANES example application, we followed workflow steps as follows:

#### Step 1. Conceptual Model Development: DAG Analysis and Covariate Selection

The data set includes 18 exposures, previously categorized in three chemical groups: 1) mono-ortho PCB 118, furans, and dioxins, 2) nondioxin like PCBs, and 3) nonortho PCBs. The LTL outcome is continuous and selected covariates include age, age,^2^ sex, race/ethnicity (non-Hispanic white, non-Hispanic black, Mexican American, other), educational attainment (less than high school, high school graduate, some college, college or more), body mass index (<25, 25–29.9, ≥30), serum cotinine, and blood cell count and distribution (white blood cell count, percent lymphocytes, percent monocytes, percent neutrophils, percent eosinophils, and percent basophils). Since this data set has been extensively described and applied elsewhere, we followed the original selection of variables and covariates outlined in Mitro et al.^39^ so we do not present a full DAG analysis here. However, we considered Weisskopf et al. caution against including all exposures and covariates in the model without careful consideration of causal structure^13^ We know from previous studies the chemicals are moderately correlated, stronger within chemical group,^5^ so expect including these moderately correlated exposures in the model is unlikely to introduce backdoor pathways. In this example scenario, we do not assume the association with each chemical is linear or that the effect of each exposure in the mixture is additive.

#### Step 2. Data Processing and Exploratory Analysis

##### Correlation of Exposures, Dimension Reduction, Statistical Power, and Missing Data

As previously noted, the correlations are moderate, ranging from 0.10 (e.g., furan to PCB) to 0.96 (e.g., within PCBs). Because the within PCB correlations were high, we may make note of this, and possibly revisit group variable selection options in models identified in Step 5 (e.g., BKMR hierarchical variable selection). We may consider an unsupervised data dimension reduction or variable selection strategy such as Bayesian Factor Analysis. We would then complete variable transformation by log-transforming and standardizing the continuous outcome, exposures, and covariates. For this example, since most of the methods we investigated do not automatically handle missing data, we could consider a complete-case analysis (i.e., removed missing data), reducing the sample size to 1,003. However, we note that any of the methods could be applied in combination with the multiple imputation approach or the strategy from Lee et al.^24^ and could be revisited in a sensitivity analysis.

#### Step 3. Study Design and Data Characteristics

This example applied cross-sectional data, with a single time point of exposure, single time point of the outcome, continuous outcome, fewer than 5,000 individuals, fewer than 20 exposures, and no requirements on the directions of effect, (Table 2). We did not consider survey or sampling weights in this scenario as the presented methods do not yet accommodate these features.

**Table 2:** Example Applications of the Mixture Method Selection Workflow

**Step**	**Question**	**NHANES Example** ^a^	**ReCHARGE Example** ^b^
**1. Conceptual Model Development**	Directed Acyclic Graph Analysis and Covariate Selection	Determined inclusion of variables	Determined inclusion of variables
**2. Data Processing and Exploratory Analysis**	Examine correlation of exposures	Moderate correlation of chemicals within groups	Low to moderate correlation across chemicals, including within chemical groups
	Exposure dimension reduction	No^c^	No
	Variable transformation	Natural log-transformed and scaled the continuous outcome (normally distributed), exposures, and covariates. Indicator terms created for covariates.	Binary outcome evaluated. Natural log-transformed and scaled the continuous exposures and covariates. Indicator terms created for categorical covariates.
	Manage missing data	Removed missing data	Removed missing data including rows with values below the LOD
**3. Study Design and Data Characteristics**	Single time point for exposure	Yes	No
	2–5 time points for exposure	No	Yes
	>5 time points for exposure	No	No
	Single time point for outcome	Yes	Yes
	2–5 time points for outcome	No	No
	>5 time points for outcome	No	No
	Spatial data	No	No
	Continuous outcome	Yes	No
	Binary outcome	No	Yes
	Categorical outcome	No	No
	Counts outcome	No	No
	Survival (time to event) outcome	No	No
	<500 individuals	No	Yes
	500 to <5 K individuals	Yes	No
	5–100 K individuals	No	No
	>100 K individuals	No	No
	<20 exposures	Yes	No
	20–100 exposures	No	Yes
	>100 exposures	No	No
	Sampling weights	No	No
	Missing data	No	No
**4. Scientific Knowledge**	Different direction of effects on outcome	No	No
	Nonlinear effects	No	No
	Prior info on groups of exposures (e.g., chemical groups)	No	No
	Prior info on individual exposures (e.g., chemical toxicity)	No	No
		**Relevant Methods Identified**	
**5. Research Questions**	Overall effect estimation	BKMR, BKMR-CMA, BMIM, BSS, ERS, FIN, MixSelect, NLInt, QGC, RH-WQS, SAID	BKMR, BSS, CWVSmix, L-WQS
	Individual exposure effects	BKMR, BKMR-CMA, BMIM, BSS, EnvMixFDR, ERS, FIN, MixSelect, NLInt, QGC, RH-WQS, SAID	BKMR, BSS, CWVSmix, L-WQS
	Interactions	BKMR, BKMR-CMA, BMIM, EnvMixFDR, FIN, MixSelect, NLInt, QGC, SAID	BKMR, CWVSmix
	Mediation	BKMR-CMA	NA^d^

a NHANES example data set: A cross-sectional 2001–2002 National Health and Nutrition Examination Survey (NHANES) data of over 1,000 adults previously described by Mitro et al.,^39^ investigating the association between exposure to 18 persistent organic pollutants (POPs) and continuous leukocyte telomere length, with covariates. This data set has been used extensively for mixtures methods testing including comparison of methods.

b ReCHARGE example data set: Follow up to the Childhood Autism Risks from Genes and Environment (CHARGE) Study (ReCHARGE) data from in the Human Health Exposure Analysis Resource (HHEAR) data repository. Case-control study of 884 children ages 2–5 years with autism spectrum disorder, nonautistic developmental delay, or typical development population controls, with information collected during pregnancy and early childhood, as described in Bennett et al.^42^ Example considered a binary ASD outcome and 83 chemical metabolites measured in urine and plasma. The analysis data set included 601 individuals with complete data on ASD, 62 chemicals, and covariates.

c “No” indicates not applied/not considered in this example application. Note, the workflow steps can be implemented multiple times for different models of interest using the same data set. Only one scenario is presented for each data set.

d NA: No methods were identified that aligned with the data set parameters and research question of interest.

#### Step 4. Scientific Knowledge

We considered the exposures to have the same direction of effect on the outcome and did not require only models to assess nonlinear effects. Mitro et al.,^39^ describe toxic equivalency factors and grouping based on chemical structure, which could be considered as prior information on chemical characteristics in modeling. However, for this example we decided not to include those features so we could first retain a larger list of methods and weigh the goals and advantages of each.

#### Step 5. Research Question

We were interested in several research questions. For the research question addressing overall effect estimation, relevant methods identified included BKMR, BKMR-CMA, BMIM, BPR, BSS, ERS, FIN, MixSelect, NLInt, QGC, RH-WQS, and SAID (Supplementary Figure 1). Because this list includes 12 methods, we could narrow it down by determining which methods are also applicable to other research questions of interest and/or whether some methods are not needed for a given context. For example, because BKMR-CMA is designed for examining mediation, we would likely not use this method because mediation analysis was not a goal or considered in the conceptual model stage of the workflow. Similarly, interactions were not of primary interest, so we could remove FIN from the considered methods. We could also revisit step 4, considering stricter goals such as including prior information on the groups of exposures and/or examining different directions of effects, and start with a smaller list (e.g., BKMR and BMIM).

#### Step 6. Assessment and Evaluation

We can then further reduce the list by considering model assumptions, performance or computational time, and model fit. In this example, we anticipate that none of the candidate methods would be computationally prohibitive for *n* = 1,003 and *p* = 20. So computational time would not be an exclusionary factor. We could consider cross-validating model fit by comparing the magnitude of training and testing errors in “out-of-sample” data. And we could examine the model residuals to determine if they are approximately normally distributed with constant variance. A researcher can also try other combinations of responses to workflow prompts to explore what other methods may work for their data and inference goals.

### Example 2. Followup to the Childhood Autism Risks from Genes and Environment (CHARGE) Study (ReCHARGE)

The longitudinal follow up to the Childhood Autism Risks from Genes and Environment (CHARGE) Study (ReCHARGE) is a case-control study of 884 children ages 2–5 years with autism spectrum disorder, nonautistic developmental delay, or typical development population controls.^42–44^ Laboratory measurements of trace elements, pesticides, phthalates, phenols, and perfluorinated chemicals or their metabolites in urine and plasma were assessed by the Human Health Exposure Analysis Resource (HHEAR) data repository.^45^ The outcome of interest was ASD phenotype, categorized as Autism Spectrum Disorder (ASD), Developmental Delay (DD), Other Early Concerns (OEC), or Typically Developing (TD).^42^ The original RECHARGE data was obtained from the publicly available data in the Human Health Exposure Resource (HHEAR) Data Repository under CHEAR project # 2016–1461; doi 10.36043/1461_222, 10.36043/1461_219, 10.36043/1461_630_2022.2.

#### Step 1. Conceptual Model Development: DAG Analysis and Covariate Selection

The original data set included 83 chemicals across five groups, as well as covariates age, sex, education, race/ethnicity, and maternal smoking during pregnancy. However, if we considered a complete-case analysis as we did for the NHANES example, the data set is reduced to 601 individuals with 62 measured chemicals. Correlations among chemicals were small to moderate as reported in Bennet et al.^42^ allowing us to apply similar reasoning as we did for NHANES and include all chemicals and covariates in our analysis.

#### Step 2. Data Processing and Exploratory Analysis

##### Correlation of Exposures, Dimension Reduction, Statistical Power, and Missing Data

As noted, correlations are small to moderate among exposures, reducing the urgency for dimension reduction. However, the dimension of 62 chemicals with repeated measurements is large enough that either some form of dimension reduction or considering a subset of the chemicals may substantially improve power. As noted above, we consider a complete-case analysis.

#### Step 3. Study Design and Data Characteristics

This example considers 2–5 time points of the exposures and a single time point of the outcome. To set up this example, we considered an analysis that examines a subset of two of the outcome categories (e.g., ASD vs TD). Accordingly, we consider a binary outcome, fewer than 500 individuals, and fewer than 100 exposures. The case-control design of the study should be considered when interpreting any analysis results for this example.

#### Step 4. Scientific Knowledge

For this example, we chose not to enforce any requirements about the same vs different direction of effects, and we do not include any prior information about groups of exposures.

#### Step 5. Research Question

We considered several research questions of interest. For overall effect estimation and individual exposure effects, we determined the same list of four methods: BKMR (using the probit model or GBKMR), BSS, CWVSmix, and L-WQS (Supplementary Figure 2). If we additionally require the inclusion of interaction effects, this list is cut down to only BKMR and CWVSmix.

#### Step 6. Assessment and Evaluation

Given the final set of methods, we can choose a final analysis approach based on additional considerations such as computational time, additional assumptions required, or model fit. For example, we could perform cross-validation with each of two methods and compare their predictive performance on out-of-sample data.

## Discussion

Informed statistical analysis of environmental mixtures data is a topic extensively explored in reviews,^3–7,9,46^ workshops,^1^ and short courses.^11^ These remain excellent guides, particularly for researchers new to the mixtures research space. We present a workflow expanding these resources by presenting a systematic strategy to approach mixtures analysis, integrating concepts from epidemiological methods, practical considerations of the data sets, scientific knowledge about the data, research questions of interest for mixtures analyses, and statistical considerations for model assessment and evaluation. Prior resources have not offered a formalized integration of epidemiological and statistical concepts for mixtures in this way. The NIEHS *GitHub* repository provides a unique space for sustainably sharing and updating available methods for mixtures analysis over time.

It is important to note that several methods may be equally appropriate for a specific context. For example, researchers may follow the workflow steps and identify four to five or more methods to consider. We do not present a comparison or contrast of methods or recommend one method over another based on a metric of performance. Rather, the workflow can be used to identify several appropriate methods for a specific scenario to educate researchers (particularly new researchers or trainees) and inform application. Subjective decision making will still be required, and careful model assessment and evaluation remain essential. We also encourage researchers to consider presenting results for more than one or two methods, thus evaluating sensitivity of the results.

In this workflow we have focused on mixture analyses differentiating between scenarios with less than 20, between 20 and 100, and over 100 exposure variables. While multiple methods considered in this workflow handle settings with over 100 variables, we do not consider the broader set of methods designed to analyze data on the exposome or other -omics data, as there are other reviews of this field available elsewhere.^34,35^ Similarly, we do not consider several important components of complex data analysis, including preprocessing steps for larger exposomic data analysis^47^ or the analysis of multiomics data.^48^ We encourage readers to also consult these and related methods for guidance on methods most suitable for large-scale ‘omic data.

The workflow considers whether a method can handle missing data or requires complete case analysis. This can be an important limitation for many contexts, where the requirement for complete case analysis can substantially reduce the final sample size. This emphasizes the importance of the first few steps considering thoughtful variable selection. Of note, the strategies themselves for performing imputation were not assessed in this paper as they were considered outside the current scope. We also acknowledge that for many methods, users interested in modifying the original source code can include modifications for handling missing data. For this paper, we only considered what is currently presented in publicly available code. Other instances where source code and data structure can be modified to accommodate a desired feature include conditional logistic regression in some methods such as quantile G-computation (QGC) using a Cox partial likelihood (https://github.com/alexpkeil1/qgcomp/). BKMR has also been modified for application to count data, accounting for spatiotemporal correlation among residuals in count models.^32^ Further, while the original implementation of WQS leveraged the assumption of directional homogeneity, recent work has sought to modify this assumption by incorporating two indices to allow associations to examine both directions of effects.^49^

Researcher preferences for index models or response surface models (see the Glossary in the Supplementary Text) are not fully incorporated in the workflow but should be considered carefully when identifying suitable methods for a given analysis. Both have distinct advantages and disadvantages, and in some ways are complementary. Linear index models reduce the dimension of the mixture into a small number, often only a single, exposure summary, which makes interpretation much more straightforward. However, these indices are often but not always formed upon strong assumptions, for example linearity and additivity of effects. If these assumptions hold, models typically provide higher statistical power to detect effects, due to model parsimony. However, if these assumptions do not hold, inference can be biased due to model misspecification. Response surfaces, in contrast, are usually modeled flexibly, which allows for the estimation of nonlinear and nonadditive effects and decreases the risk of model misspecification. However, this flexibility requires more data, especially in higher exposure dimensions, and can have lower power than index methods when the simpler assumptions hold or nearly hold.

We characterize whether the presented methods can include weights, such as survey or oversampling weights. However, methods taking weights into account are not well represented in the mixtures literature and warrant further development. We also did not present methods specific to modification by factors that are not members of the mixture. This can theoretically be addressed by any of the methods through stratification or subsetting the data if the number of subgroups is small, although in certain scenarios there may be more statistically efficient methods that pool some information across groups (e.g., interaction analyses that assume the effects of exposures vary by subgroup, but the effects of covariates do not vary by subgroup).

We organize the workflow in sequential steps. However, we recognize some researchers may wish to first consider the research question (Step 5) before developing a conceptual model (Step 1). The benefit of the online tool is that a researcher can select multiple features at once to identify relevant methods (e.g., continuous outcome, multiple time points of exposure, single time point of outcome, fewer than 500 individuals, assumes same direction of effect, etc.). So, although the workflow is organized in sequential steps to guide the reader through each conceptual area, the online interface will operate more as an advanced search tool, where a user can select multiple features of interest and then click “search” to identify relevant methods. Similarly, Step 6 is intended to occur after implementing models, aligned with evaluating model fit. However, researchers can also consider these details prior to model implementation as known limitations such as convergence issues or computation time may impact the selection of models.

We also note the potential to apply two mixtures methods sequentially, particularly unsupervised and supervised strategies. This can be a helpful way to approach analysis of high dimensional data sets when interested in applying a method not well suited for big data. For example, in step 2 of the workflow, an exposure data dimension reduction step can be applied to reduce a high number of exposure variables of interest to a smaller set of clusters or components, especially if interest lies in exposure pattern recognition. Then researchers may leverage methods identified in Step 3 suitable for data sets with 2 – 5 exposure patterns. Researchers must also consider the model assumptions of both methods applied. Another example is the use of penalized likelihood, particularly the elastic net, to form an exposure index, known as an environmental risk score, on a portion of the data, that can then be tested in the remaining data.^50^

A unique strength of this work is the development of a publicly available *GitHub* repository listing mixtures methods discussed in this paper, characterized for each step in the workflow. The repository table is designed to evolve over time and can highlight important research gaps in existing mixtures methods and/or the need for potential extensions of approaches to include other complex data such as larger scale exposomics or multiomics. Additional training resources and methods development to address precision environmental health, causal methodology, risk assessment, and mediation approaches are important areas warranting further research and integration in this resource.

## Supplementary Material



## Notes

Glossary of terms, table summarizing workflow steps 1–6, and figures showing the application of workflow steps 3–5 (DOCX)
