# Supplementary material for: Workflow for Statistical Analysis of Environmental Mixtures
Source: Environ Health Perspect. 2026 Apr 19;134(1):8–22. doi: 10.1021/EHP.6c00155 (PMC13151037; doi:10.1021/EHP.6c00155)
Supplement: Supplementary file 1 [file hp6c00155_si_001.docx]

**Supplemental Text and Tables**

# Glossary

## Co-exposure amplification bias

Unmeasured confounding can be problematic in mixtures epidemiology. Models that include many environmental exposures can potentially amplify the amount of bias, known as co-exposure amplification bias, depending on the structure of unmeasured confounding, relative to single exposure models. The amount of bias depending on the correlation among the exposure measures and the strength of the unmeasured confounding.

## Out-of-sample predictive performance

General term for the accuracy of a fitted model’s predictions on data not used to fit the model. Accurate out-of-sample predictions suggest the model has found a true statistical relationship in the data, rather than simply being “overfit” to the particular observations it was trained on.

## MCMC convergence

A Markov Chain Monte Carlo (MCMC) algorithm for Bayesian model fitting has converged when it is generating samples from the posterior distribution of the model parameters.

## Index model for supervised methods

A multi-exposure index is a weighted sum of exposure variables, meant to represent exposure to the mixture of exposures. In supervised index models, the weights of the index are estimated within a health effects model for an outcome, thereby estimating a weighted average most associated with the outcome. Popular examples of index models for environmental mixtures included weighted quantile sum regression and quantile G-computation, with Bayesian multiple index models being a relatively new class of models.

## Response surface model

Characterizes the exposure-response relationship by a multi-dimensional surface that represents how the mean of an outcome varies for any given value of the exposures. Because this surface is often estimated non-parametrically (that is, with minimal assumptions on the shape or structure of this surface), this class of models often allows for estimation of non-linear and non- additive relationships between exposures and response, thereby allowing for a broad array of exposure-response relationships. Examples in environmental mixtures analyses include multivariate generalized additive models, kernel machine regression, and MixSelect.

## Latent factors (latent variable model)

A latent variable model assumes that the joint distribution of observed variables (sometimes referred to as “manifest variables”) can be represented by a smaller number of latent, unobserved, factors. In environmental mixture studies, a latent variable model typically assumed latent variables that generate the joint distribution of multiple exposures and the outcome. Examples include Factor Analysis for Interactions (FIN) and Bayesian profile regression.

## Distributed lag model

A distributed lag model characterizes how an outcome depends on a sequence of values of an exposure. While original applications in the environmental health sciences focused on time series of outcomes, such as how daily mortality counts are associated with daily air pollution exposures up to two weeks (say) prior to the date of death, this class of models has also be popular in children’s health studies focusing on developmental windows of susceptibility to an environmental exposure during pregnancy and childhood. In this context, distributed lag models for environmental mixtures include BKMR-DLM, structured Bayesian regression tree pairs, and multiple exposure distributed lag models with variable selection.

# Supplementary Table 1. Summary of Workflow Steps 1 – 6

| **Workflow Step** | **Approach(es)** | **Description/Key Questions** |
| --- | --- | --- |
| 1. **Conceptual Model Development** | Directed Acyclic Graph Analysis and Covariate Selection | Hypothesize the causal relationships between the outcome and exposures considered to be part of the mixture. Identify covariates to include as modifiers or confounders, considering potential biases and research question(s). Note, some steps below may modify this initial list of variables to include in the model (e.g. further reduce the list). |
| **2. Data Processing and Exploratory Analysis** | Examine correlation of exposures | Highly correlated exposure variables may arise from the same source and travel together, making it difficult to untangle individual exposure effects. Consider exposure sources and exposure contrasts in the dataset. This can impact the final list of variables to include the in model. |
|  | Examine statistical power | Investigate the probability that a given method will detect an association given assumed data generation model, sample size, and effect size. |
|  | Exposure dimension reduction (optional) | Consider reducing highly corelated exposure data depending on exposure data characteristics. |
|  | Variable transformation (optional) | Transform and/or scale the outcome, exposure, and/or covariate variables. Standardizing exposure data to the same scale is typically recommended. Log-transforming continuous outcomes to follow normal distributions can be important for some of the latent variable methods, depending on model assumptions (for such cases, review model residuals). |
|  | Manage missing data | Consider missing values due to values below the limit of detection (LOD) or outright missingness, and strategies for imputation. |
| **3. Study Design and Data Characteristics** | Single or repeated timepoint for exposures and outcomes | Is the study longitudinal, with repeated measurements of exposures and/or outcomes or is there only a single timepoint for the measurement of exposure and outcome variables? |
|  | Spatial data | Does the study include data with spatial variation and/or does spatial correlation among outcomes need to be considered in the model? |
|  | Distribution of the outcome | Does the dataset include a continuous, binary, categorical, count, or time-to-event (survival) outcome variable? |
|  | Size of the dataset | How many individuals and how many exposures/variables are included in the dataset for analysis? |
|  | Survey or sampling weights | Are there survey or sampling weights to include in the analysis? |
| **4. Scientific**  **Knowledge** | Expected effects of exposures within a mixture on the outcome | Utilize existing information from the literature, preliminary data, and/or toxicology to inform the expected effect of the exposure on the outcome. Are exposures hypothesized to act in the same direction, or should the model allow for the possibility effects operate in different directions? Is the exposure-response relationship likely to be non-linear? Is there biological, toxicological, or other information about the potential effects of the exposures such as chemical groups that should be included in the statistical model? Are there chemical properties/features to include in the model?” |
| **5. Research Questions** | Overall effect estimation | What is the research question of interest for this analysis?  Do you wish to determine the overall or aggregate effect of the mixture of exposures on a health outcome? |
|  | Individual exposure effects | Do you wish to identify independent effects of mixtures components (“toxic agents” or “bad actors”)? |
|  | Interactions | Do you wish to allow potential interaction effects among mixture components? |
|  | Mediation | Do you wish to examine either the role of a mediator on the pathway between a mixture and an outcome, or the role of a mixture as a mediator on the pathway of another risk factor and an outcome? |
| **6. Assessment and Evaluation** | Assumptions | Examine the underlying assumptions of each model identified relevant to a scenario (e.g., multivariate normal distribution, constant variance) |
|  | Convergence | Confirm successful model convergence (e.g., by using trace plots, MCMC model output, etc.) |
|  | Overfitting | Assess model results for fit and performance (e.g., evaluate “out-of-sample” data) |

# Supplementary Figure 1. Application of the Workflow Steps 3-5: NHANES Example Data

Workflow responses for the NHANES example scenario are in row 1; methods that can be applied given those responses are displayed in row 2 and below.


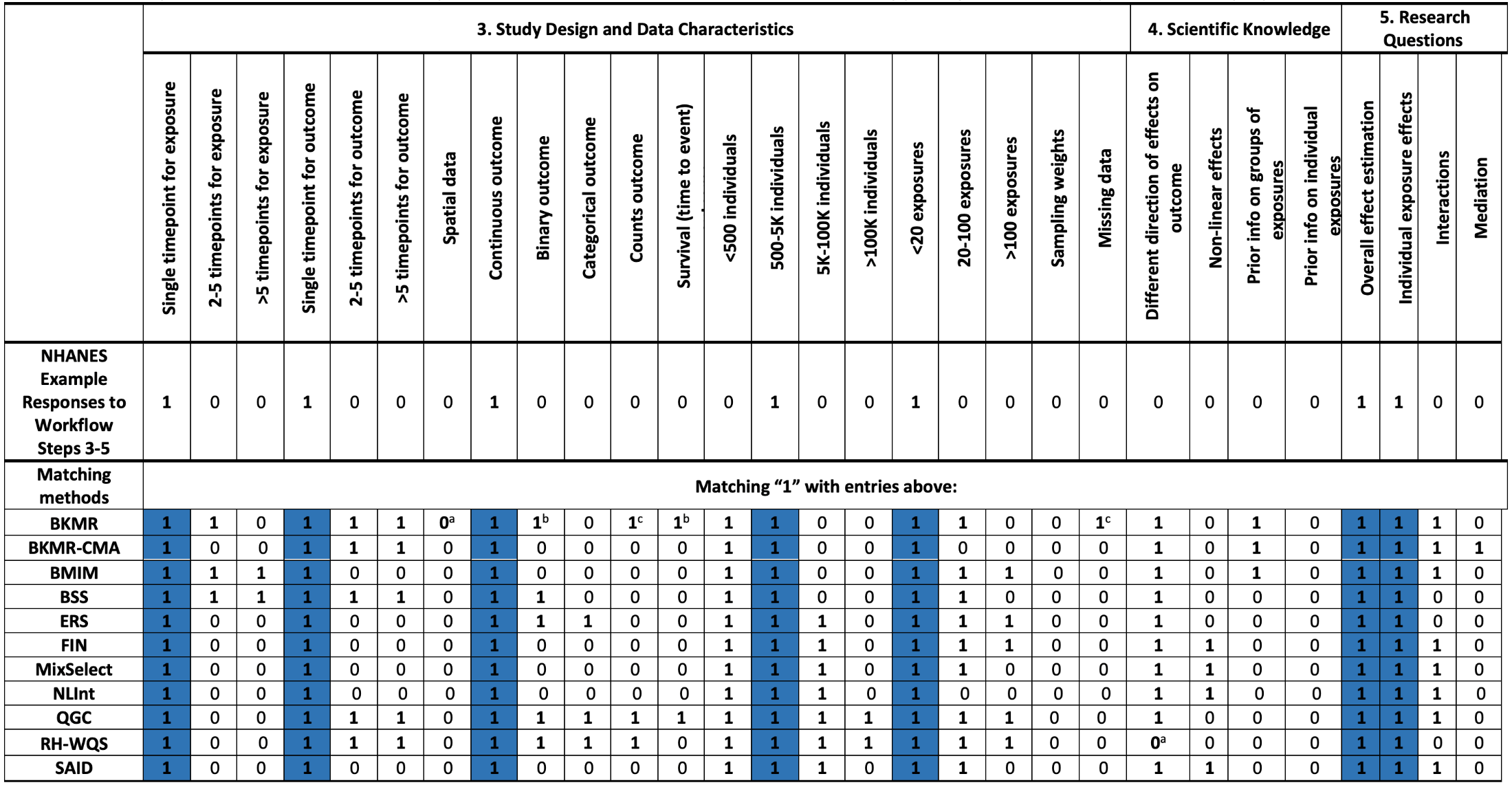


*NHANES example dataset: A cross-sectional 2001-2002* *National Health and Nutrition Examination Survey (NHANES) data of over 1,000 adults previously described by Mitro et al. ^39^, investigating the association between exposure to 18 persistent organic pollutants (POPs) and continuous leukocyte telomere length, with covariates. This dataset has been used extensively for mixtures methods testing including comparison of methods. Note, the workflow steps can be implemented multiple times for different models of interest using the same dataset. Only one scenario is presented.*

*0=No/1=Yes responses reflect how the method was described in the original publication or in publications applying the method in an epidemiological dataset and where publicly available code with documentation (e.g. an R package) for this type of application is available.*

*0^a^: It is possible to manipulate the source code to align with this feature (e.g., using a cox proportional hazards partial likelihood to perform conditional logistic regression), but the code is not currently publicly available.*

*1^b^: The method can be applied but may have lower performance/speed or interpretability compared to other methods.*

*1^c^: This method can be applied with publicly available code that is outside the main methods package.*

# Supplementary Figure 2. Application of the Workflow Steps 3-5: ReCHARGE Example Data

Workflow responses for the ReCHARGE example scenario are in row 1; methods that can be applied given those responses are displayed in row 2 and below.


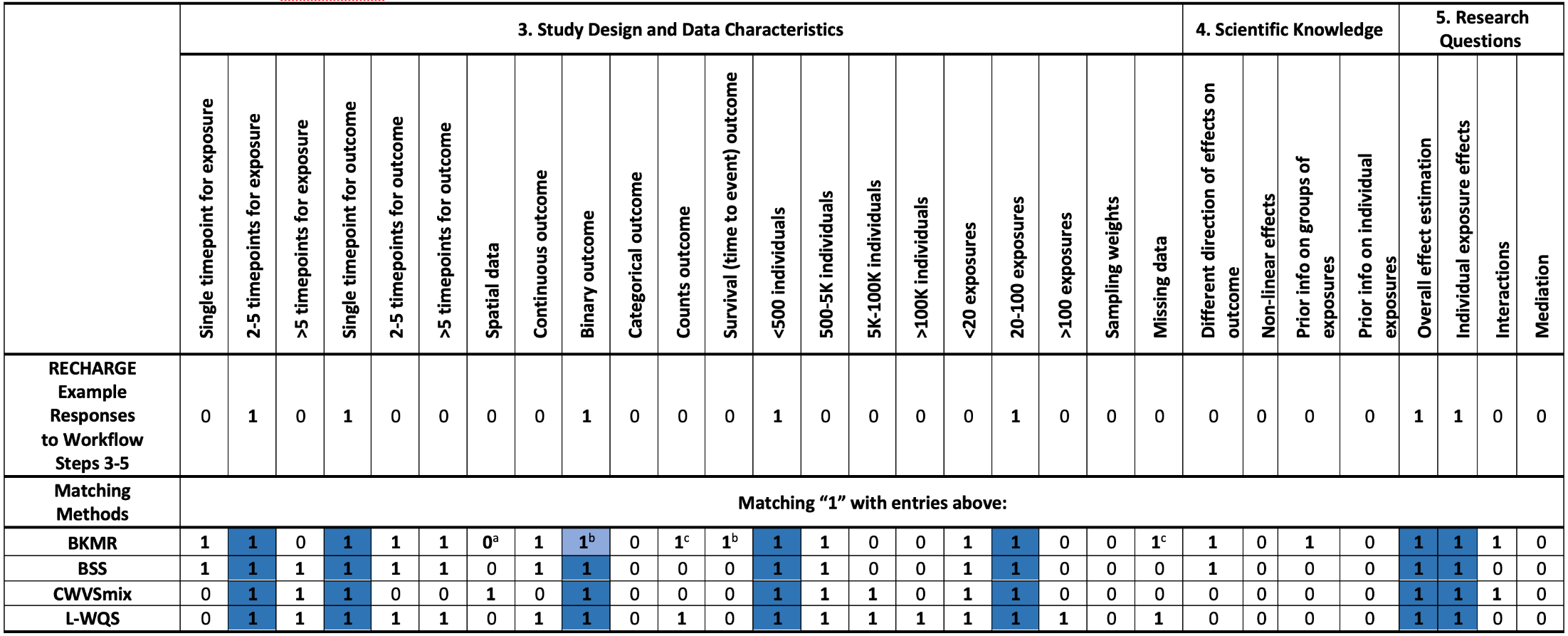


*ReCHARGE example dataset: Follow up to the Childhood Autism Risks from Genes and Environment (CHARGE) Study (ReCHARGE) data from in the Human Health Exposure Analysis Resource (HHEAR) data repository. Case-control study of 884 children ages 2-5 years with autism spectrum disorder, non-autistic developmental delay, or typical development population controls, with information collected during pregnancy and early childhood. Example considered a binary ASD outcome and 83 chemical metabolites measured in urine and plasma. The analysis dataset included 601 individuals with complete data on ASD, 62 chemicals, and covariates. Note, the workflow steps can be implemented multiple times for different models of interest using the same dataset. Only one scenario is presented.*

*0=No/1=Yes responses reflect how the method was described in the original publication or in publications applying the method in an epidemiological dataset and where publicly available code with documentation (e.g. an R package) for this type of application is available.*

*0^a^: It is possible to manipulate the source code to align with this feature (e.g., using a cox proportional hazards partial likelihood to perform conditional logistic regression), but the code is not currently publicly available.*

*1^b^: The method can be applied but may have lower performance/speed or interpretability compared to other methods.*

*1^c^: This method can be applied with publicly available code that is outside the main methods package.*
